# Supplementary material for: Empirical methods for controlling false positives and estimating confidence in ChIP-Seq peaks
Source: BMC Bioinformatics. 2008 Dec 5;9:523. doi: 10.1186/1471-2105-9-523 (PMC2628906; doi:10.1186/1471-2105-9-523)
Supplement: Additional File 1 — A variety of html documents from the USeq web site detailing the available applications, their best usage, output file type descriptions, command line menus, etc. [file 1471-2105-9-523-S1.zip › USeqUserGuides/JavaDoc.zip!/JavaDoc/expr/package-frame.html]

expr


expr

Classes 
  
CorrelationMaps
  
CorrelationWindow
  
ExpressedGene
  
ExpressionScores2CoordinatesViaGFF
  
FileCrossFilter
  
FileMatchJoiner
  
MiRNA
  
MiRNATarget
  
NormalizeAgilentData
  
ParseAgilentData
  
ParseManipulateColumnDataForGeneSifter
  
ParseMiRNATable
  
ParseWormMart
  
StripZeros
